# Seed Banks as Incidental Fungi Banks: Fungal Endophyte Diversity in Stored Seeds of Banana Wild Relatives

**DOI:** 10.3389/fmicb.2021.643731

**Published:** 2021-03-22

**Authors:** Rowena Hill, Theo Llewellyn, Elizabeth Downes, Joseph Oddy, Catriona MacIntosh, Simon Kallow, Bart Panis, John B. Dickie, Ester Gaya

**Affiliations:** ^1^Department of Comparative Plant and Fungal Biology, Royal Botanic Gardens, Kew, Richmond, United Kingdom; ^2^School of Biological and Chemical Sciences, Faculty of Science and Engineering, Queen Mary University of London, London, United Kingdom; ^3^Department of Life Sciences, Faculty of Natural Sciences, Imperial College London, London, United Kingdom; ^4^Department for Environment, Food and Rural Affairs, London, United Kingdom; ^5^Department of Plant Science, Rothamsted Research, Harpenden, United Kingdom; ^6^School of Life Sciences, University of Glasgow, Glasgow, United Kingdom; ^7^Collections Department, Royal Botanic Gardens, Kew, Millennium Seed Bank, Ardingly, United Kingdom; ^8^Division of Crop Biotechnics, Department of Biosystems, Faculty of Bioscience Engineering, University of Leuven, Leuven, Belgium; ^9^Bioversity International, Montpellier, France

**Keywords:** endophytic fungi, seed banking, seed mycobiome, banana, crop wild relatives, *Musa*, *Fusarium*

## Abstract

Seed banks were first established to conserve crop genetic diversity, but seed banking has more recently been extended to wild plants, particularly crop wild relatives (CWRs) (e.g., by the Millennium Seed Bank (MSB), Royal Botanic Gardens Kew). CWRs have been recognised as potential reservoirs of beneficial traits for our domesticated crops, and with mounting evidence of the importance of the microbiome to organismal health, it follows that the microbial communities of wild relatives could also be a valuable resource for crop resilience to environmental and pathogenic threats. Endophytic fungi reside asymptomatically inside all plant tissues and have been found to confer advantages to their plant host. Preserving the natural microbial diversity of plants could therefore represent an important secondary conservation role of seed banks. At the same time, species that are reported as endophytes may also be latent pathogens. We explored the potential of the MSB as an incidental fungal endophyte bank by assessing diversity of fungi inside stored seeds. Using banana CWRs in the genus *Musa* as a case-study, we sequenced an extended ITS-LSU fragment in order to delimit operational taxonomic units (OTUs) and used a similarity and phylogenetics approach for classification. Fungi were successfully detected inside just under one third of the seeds, with a few genera accounting for most of the OTUs–primarily *Lasiodiplodia*, *Fusarium*, and *Aspergillus*–while a large variety of rare OTUs from across the Ascomycota were isolated only once. *Fusarium* species were notably abundant–of significance in light of Fusarium wilt, a disease threatening global banana crops–and so were targeted for additional sequencing with the marker *EF1α* in order to delimit species and place them in a phylogeny of the genus. Endophyte community composition, diversity and abundance was significantly different across habitats, and we explored the relationship between community differences and seed germination/viability. Our results show that there is a previously neglected invisible fungal dimension to seed banking that could well have implications for the seed collection and storage procedures, and that collections such as the MSB are indeed a novel source of potentially useful fungal strains.

## Introduction

Fungal endophytes (hereafter, endophytes) are fungi which live asymptomatically inside plant tissues (Petrini, 1991; Hardoim et al., 2015), and appear to be present in all land plants (Stone et al., 2000; Rodriguez et al., 2009; Rashmi et al., 2019). Certain endophytes are known to provide various benefits to the plant host, such as stress tolerance, growth promotion, and disease resistance (Redman et al., 2002; Waller et al., 2005; Bilal et al., 2018). As a result, endophytes have become of particular interest in agriculture due to their promise as pest and pathogen biocontrol agents, ideally replacing and/or reducing ecologically harmful chemical controls (Card et al., 2016; Bamisile et al., 2018; Vega, 2018) and aiding sustainable intensification of agriculture without increased use of chemical fertilisers (Le Cocq et al., 2016). Additionally, they can produce a suite of secondary metabolites as part of the plant-fungal interaction, providing a valuable opportunity for discovery of new therapeutically relevant bioactive compounds, such as antivirals and antibiotics (Gupta et al., 2020). Since endophytes are likely to comprise a considerable proportion of the more than 2 million estimated species of yet undiscovered fungi (Petrini, 1991; Arnold et al., 2000; Hawksworth and Lücking, 2017), capitalising on these potentially useful fungi requires continued efforts toward discovering endophyte diversity.

Collections are an important resource for biodiversity research, as they provide curated specimens spanning time and space (Funk, 2018; Pearce et al., 2020). Beyond prospecting for novel fungal diversity in dedicated mycological collections such as fungaria and culture collections (Dentinger et al., 2016; Andrew et al., 2018, 2019; Huang et al., 2018), we now recognise the potential for plant collections to be a source of fungal diversity too. Daru et al. (2018) showed that sequencing dried herbarium specimens can reveal novel endophyte diversity, with the caveat that it is extremely rare to recover viable endophytes in culture. Compared to herbaria, seed banks (preserved seed collections) are an alternative with the considerable advantage of being living collections, enabling isolation of live fungal strains in culture. This is particularly valuable as it facilitates the compilation of endophytic culture collections for further study, such as in inoculation experiments to identify strains that are beneficial to plant health (e.g., Waller et al., 2005; Bilal et al., 2018) or whole-genome-sequencing to investigate the genomic basis of the plant-endophyte interaction (e.g., Zuccaro et al., 2011; Niehaus et al., 2016; Knapp et al., 2018). Nevertheless, there is far less known about seed endophytes compared to those in leaves, the latter of which may be more abundant (Bayman et al., 1998; Ganley and Newcombe, 2006) although few studies have looked at both seed and foliar endophytes for the same host plant, and to our knowledge always in the wild.

Seed banks were initially conceived in the 20th century as a measure to conserve crop genetic diversity (Peres, 2016), the most famous example likely being the Svalbard Global Seed Vault (Westengen et al., 2013). The Millennium Seed Bank (MSB), managed by the Royal Botanic Gardens Kew, is the world’s largest seed bank and part of a global partnership network for seed conservation (Liu et al., 2020). The MSB is notably directed to wild plant conservation, with one of its priorities being crop wild relatives (CWRs) (Liu et al., 2018). CWRs, the close relatives of our domesticated crop species, act as an additional pool of genetic diversity to breed improvements into our crops, such as increased productivity and resilience against disease and environmental stressors (Hajjar and Hodgkin, 2007; Brozynska et al., 2016). More recently, similar benefits have been equally demonstrated by inoculation of various crops with endophytes from CWRs (Murphy et al., 2018a, b, 2019). This potential role of CWR endophytes in both the health of wild plant populations and their crop counterparts brings in additional value to the MSB collections, making them not only important for plant conservation, but also plant microbiome conservation.

The role of endophytes in plant health is, however, more complicated than it first seems. Despite their ubiquity, the endophytic lifestyle remains somewhat of a mystery. While there are aforementioned examples of mutualistic endophytes found only to benefit the plant host, other fungi described as endophytes are found to be latent pathogens or decomposers (saprotrophs) (Slippers and Wingfield, 2007; Promputtha et al., 2010; Delaye et al., 2013; Nelson et al., 2020). This concept–that the term endophyte represents a range of functional roles within the plant host–has been referred to as the *endophytic continuum* (Schulz and Boyle, 2005). Of the 366 species classified as endophytes in the FUNGuild database as of December 2020: 140 (∼38%) are also classified as plant pathogens; 16 as saprotrophs (∼4%); and 17 (∼5%) as other various guilds (Nguyen et al., 2016). Indeed, in phylogenetic analysis, endophytes are commonly found to be closely related to pathogens and saprotrophs, as well as endolichenic fungi, their lichen-associated counterpart (Arnold et al., 2009; U’Ren et al., 2009, 2010). A habit switch from commensal to pathogenic has been observed in some endophytes due to unfavourable environmental conditions (Slippers and Wingfield, 2007; Ribeiro et al., 2020), and there is evidence that endophytes found only in living tissues don’t significantly differ in cellulolytic activity (i.e., decomposing capacity) from those found only in dead leaves (U’Ren and Arnold, 2016). Together, these evoke the obscure position of endophytes on the mutualistic-commensal-pathogenic spectrum and highlights our ignorance regarding their nutritional strategies.

Consequently, there is ambiguity as to which endophytes inhabiting stored seeds are beneficial–or even essential–to the plant host, and which are potentially harmful. This uncertainty has obvious implications in seed storage protocols, which most often focus on the harmful fungi. For example, in internationally recognised reports on best-practise gene banking, mention of fungi (and bacteria) is almost exclusively in the context of avoidance, with recommendations for the use of antifungals/antibiotics on collections (Food and Agriculture Organization, 2014; Center for Plant Conservation, 2018). These recommendations overlook an essential question: what is the impact of not preserving healthy endophyte communities when banking seeds? How do endophyte communities impact the success of recovered plant populations down the line? Such endophytic communities may be playing similar roles as the microbial associates of humans or animals, which we now know to be essential for normal, healthy functioning and imbalances of which cause disease (Dudek-Wicher et al., 2018). While great care is taken to optimise the phylogenetic and geographical diversity and longevity of MSB seed collections, consideration of the microbial communities associated with the seeds is notably absent (Liu et al., 2020). Considering that there are endophytes known to be implicated in germination and seedling success (Tamura et al., 2008; Hubbard et al., 2014; Li et al., 2017; Shearin et al., 2018; Leroy et al., 2019), this is a significant oversight.

To explore these issues and demonstrate the value of seed banks for endophyte discovery, we focused on a case study of CWRs of banana (and plantain, *Musa* spp. L.)–one of the most important crops in the world. Global production of banana is estimated to be 116 million tonnes annually, worth $31 billion (Food and Agriculture Organization, 2020b). *Musa* taxa are tall herbaceous monocarpic monocotyledons in the family Musaceae, order Zingiberales. They are native to tropical and subtropical Asia to western Pacific regions (Govaerts and Häkkinen, 2006) with approximately 80 taxa (hereon called “species”) in the genus (Häkkinen and Väre, 2008; POWO, 2019). There are around 1,000 cultivars of edible bananas (Ruas et al., 2017; Food and Agriculture Organization, 2020a), most of which stem from two species: *Musa acuminata* Colla and *Musa balbisiana* Colla (Carreel et al., 2002; Langhe et al., 2009; Perrier et al., 2011; Rouard et al., 2018; Martin et al., 2020). In spite of this diversity, the vast majority of commercial banana plantations are clones of a single cultivar, Cavendish, which makes the crop highly susceptible to disease (Ordonez et al., 2015). In the 1970s, *Fusarium oxysporum* f. sp. *cubense* emerged to cause Fusarium Wilt of Cavendish bananas, and has since spread across the global tropics to most banana producing countries^1^ (Dita et al., 2018). The predominant strain is commonly referred to as Foc TR4, and was recently proposed to belong to the novel species *Fusarium odoratissimum* (Maryani et al., 2019). Considering the global value of the banana crop, 85% of which is eaten locally as a major contribution to people’s diets (Food and Agriculture Organization, 2020b), Foc TR4 represents a major threat to both economic and food security in banana producing countries. Stored banana CWR seeds are a precious conservation resource in light of the susceptible Cavendish banana cultivar, and so present a valuable case-study for investigating associated endophyte diversity.

While many endophytic species can be grown in culture, many more cannot, and so molecular tools are relied upon to detect much more of the true extent of endophytic diversity (e.g., Higgins et al., 2011; Parmar et al., 2018; U’Ren et al., 2019). Nonetheless, culturing is still a necessary tool, as it not only isolates strains for future study, but also provides an indication of which fungal strains are alive, which is particularly relevant when assessing post-storage endophytes. Here we used both a culture-dependent and culture-independent approach to maximise discovery of endophytic diversity from accessions belonging to six species of banana wild relatives in the genus *Musa*. By PCR-cloning individual seed DNA extractions for the culture-independent approach, we were able to assess the number of unique operational taxonomic units (OTUs)–a proxy for species–per seed. We made use of metadata and seed viability assessments from the MSB collections in order to explore the association of habitat, host *Musa* species, post-storage seed viability and germination rate with endophyte community composition, diversity and abundance.

## Materials and Methods

Seeds from 45 *Musa* accessions (with each accession containing 50 seeds collected from between one and five plant individuals belonging to the same *Musa* species in the sampling site) were obtained from the Millennium Seed Bank (MSB), all of which had been stored at −20°C. Seeds had been collected in 15 localities in Vietnam and Malaysia, with one accession collected from RBG Kew’s living collections (see Supplementary Table 1 for details). For each accession, seeds were randomly split into batches of 20 for fungal culturing, 10–20 for direct sequencing and 60 for seed viability assessment.

### Seed Viability Assessment

Post-storage seed viability was assessed using two methods. Firstly, the tetrazolium chloride test (TTC) was carried out following the approach of Leist and Krämer (2011). Seeds were imbibed on agar for 3 days at 20°C. Then a proportion of the testa was removed using a scalpel on two lateral sides to expose the endosperm. Seeds were then soaked in 1% buffered 2,3,5-triphenyl tetrazolium chloride (pH6-8) for 2 days at 30°C in the dark. Staining patterns were recorded. Embryos that completely stained dark red, or that showed dark red staining at the embryonic axis (the opposite from the haustorium) were considered viable; light pink staining or white embryos were considered unviable. Fifty seeds per accession were tested.

The second viability test was embryo rescue (ER). In a laminar flow, seeds were sterilised by soaking them in 96% ethanol for 3 min, followed by 20% bleach (NaOCl containing 1 drop of detergent per 100 ml) for 20 min, then seeds were rinsed three times in sterilised water. Continuing in the laminar flow with sterile forceps and scalpel, embryos were extracted from seeds. This was done using an incision in the seed coat next to the micropyle and manipulating the seed in order to split the testa; the embryo was then gently removed. Embryos were subsequently transferred onto autoclaved half MS medium (Murashige and Skoog, 1962) in tubes using long forceps with the haustorium in contact with the medium and the embryonic axis upward. Tubes containing embryos were incubated in the dark at 27°C for 14 days after which they were put in a growth chamber in the light at 27°C for an additional 14 days. Six possible observations were recorded: shoot, callus, blackened colouration, no embryo, contamination, no change. Ten seeds per accession were tested.

### Surface Sterilisation and Isolation of Fungal Cultures

Following the protocol of Arnold et al. (2007), seeds were surface sterilised by sequential immersion in 95% ethanol (10 s), 10% bleach (2 min), and 70% ethanol (2 min), then left to surface-dry under sterile conditions. For the culture-dependent approach 20 sterilised seeds per accession were plated on 2% malt extract agar (MEA), sealed with parafilm and incubated at room temperature. Emergent fungal growth was transferred to a new plate for pure culture isolation. Additionally, imprints of sterilised and unsterilised seeds were made on 2% MEA to confirm the efficacy of the sterilisation methodology, and to observe whether the same species found inside the seeds were also present externally on the unsterilised seed surface. Vouchers of culture isolates were cryopreserved and deposited at RBG Kew. Cryopreservation involved transferring ∼four 5 mm^2^ squares of agar into each of two 2 ml tubes containing 1 ml of sterile 10% glycerol. Tubes were brought from room temperature to −80°C at the rate of 1°C per minute to prevent shock.

### DNA Extraction and Sequencing

For the axenic cultures, DNA was extracted using the Extract-N-Amp Plant PCR Kit (Sigma-Aldrich, St. Louis, MO, United States) in 5 μl of extraction buffer and 5 μl of dilution buffer. For the culture-independent approach, 10–20 seeds were surface-sterilised as described above and individually transferred to sterile 2 ml collecting tubes, frozen at −80°C and pulverised using a Mixer Mill MM 400 (Retsch, Germany) with two sterilised stainless-steel beads per tube. The Qiagen DNeasy Plant Mini Kit (Qiagen, Redwood City, CA, United States) was used for direct extraction of the pulverised seeds according to the manufacturer’s protocol, with DNA eluted in 100 μl of TE buffer.

Extract-N-Amp PCR ReadyMix was used for amplification of both culture and direct extractions. The total PCR reaction volume was 8 μl, consisting of 0.5 μl of extracted DNA, 4 μl Extract-N-Amp PCR ReadyMix, 3.42 μl of distilled water and 0.4 μl of each primer (100 μM). The primers used to amplify the ITS-partial LSU rDNA fragment were ITS1F (Gardes and Bruns, 1993) and LR3 (Vilgalys and Sun, 1994). Cycling conditions were as follows: 94°C for 3 min, 35 amplification cycles (94°C for 30 s, 53°C for 35 s, 72°C for 1 min, with the addition of 5 s to the extension phase per cycle), and 72°C for 4 min.

To account for the possibility of multiple species of fungal endophyte per seed, PCRs of direct extractions were cloned into the pCR^TM^4-TOPO^®^ *Escherichia coli* vector with the TOPO TA-Cloning Kit (Invitrogen, Carlsbad, CA, United States) according to the manufacturer’s instructions. Up to 12 colonies were randomly picked and amplified in secondary PCR with the above cycle conditions and primers.

After examination with gel electrophoresis (1% agarose in TE buffer), all PCR products were purified using ExoSAP-IT (USB, Hudson, OH, United States) and both strands sequenced using the same primers as for amplification. Sequence reactions were carried out in 5 μl volumes–consisting of 1 μl 5× sequencing buffer, 2.05 μl ddH_2_0, 1 μl primer (1.25 μM), 0.5 μl purified PCR product and 0.45 μl BigDye Terminator v3.1 (Applied Biosystems, Foster City, CA, United States)–and run on an ABI 3730 DNA Analyzer (Applied Biosystems, Foster City, CA, United States). Sequences were manually edited with contiguous alignments using Geneious R7 v7.1.5 (Biomatters, New Zealand). Sequences from 642 endophytes (235 cultures, 280 direct sequences, and 127 clones) have been deposited in GenBank under accession numbers MW298868-MW299510.

### OTU Delimitation and Taxonomic Identification

Sequences were clustered into OTUs using the *de novo* method USEARCH v10.0.240 as part of the UPARSE pipeline (Edgar, 2013). As USEARCH is sensitive to fragments of different length, ITSx (Bengtsson-Palme et al., 2013) was used prior to clustering to extract the 5.8S and ITS2 regions–shown to recover more fungal OTUs when used together (Heeger et al., 2019)–while LSU fragments were manually trimmed to the same length after alignment with MUSCLE v3.8.425 (Edgar, 2004) and visualisation in AliView v1.17 (Larsson, 2014). Dereplication was performed via removal of identical sequences using the fastx_uniques functions inbuilt to USEARCH. 5.8S-partial LSU OTUs were clustered using a 99% similarity threshold, guided by the optimal threshold for species discrimination using ITS/LSU identified by Vu et al. (2019). Singletons–OTUs comprising one sequence–were not discarded, as is common practise to reduce artefacts when using NGS datasets, because each sequence originated from Sanger sequencing of an individual seed extraction, and so was assumed to be “real.”

Preliminary identification of OTUs was made via a local BLASTn v2.6.0 search (Camacho et al., 2009) against the UNITE v8.2 database, release 04.04.2020 (Abarenkov et al., 2020). Taxonomic identification of OTUs was inferred from the top UNITE hit, guided by Vu et al. (2019): ≥99% similarity for the same species; ≥98 similarity for the same genus; ≥96 similarity for the same family; ≥94 similarity for the same order; ≥92 similarity for the same class; and <92% similarity for the same phylum. Similarity-based identification was corroborated with a phylogenetic approach via the Tree-Based Alignment Selector (T-BAS) toolkit v2.2 (Miller et al., 2010; Carbone et al., 2019), a platform designed for preliminary placement and visualisation of unknown fungal sequences in curated multilocus phylogenies. Representative sequences for 181 OTUs were placed in the 6-loci *Pezizomycotina* v2.1 and the 6-loci Fungi reference trees (James et al., 2006; Carbone et al., 2017) with default settings and using the evolutionary placement algorithm (EPA) option from RAxML (Berger et al., 2011; Stamatakis, 2014). OTU taxon assignment was altered to reflect the lowest taxonomic level in agreement between both T-BAS and UNITE, with the UNITE species level identification used if T-BAS and UNITE agreed on genus and the UNITE percentage identity was ≥99%. All filtering of classification data was done using R v3.5.3 in RStudio v1.1.463 (RStudio Team, 2015; R Core Team, 2020), the script for which is available at https://github.com/Rowena-h/MusaEndophytes.

### Sampling Effort and Community Analysis

For the purpose of these analyses, *Musa* subspecies and varieties were grouped under the same species. Sampling effort was assessed by producing species accumulation curves of the number of OTUs for the number of *Musa* accessions using the rarefaction method in the specaccum function from the R package vegan v2.5-6 (Oksanen et al., 2019). This was done including and excluding singleton OTUs for all *Musa* accessions (*n* = 45) as well as distinguishing between the three best sampled species–*M. acuminata* (*n* = 12), *M. balbisiana* (*n* = 16), and *Musa itinerans* (*n* = 14). The impact of detection method–culturing, direct sequencing or cloning–on species recovery was quantified with analysis of similarity (ANOSIM) (Clarke, 1993) using the vegan anosim function following confirmation that data dispersion was even using the vegan betadisper function.

The RBG, Kew accession and oil palm plantation accessions (1 locality in Malaysia) were excluded from the following analyses due to low sample size for the habitats and the former being a geographical outlier. Endophyte community composition was explored using non-metric multidimensional scaling (NMDS) implemented in the metaMDS function in vegan. OTU counts were filtered for the eight most common OTUs (abundance greater than 20) for the 33 accessions of *M. acuminata*, *M balbisiana*, and *M. itinerans* and six dimensions were selected for the NMDS using a scree plot (Supplementary Figure 1). Habitat information for *Musa* accessions was interpreted from the collection notes in the MSB’s metadata records (Supplementary Table 1). To investigate the relationships between community composition and post-storage seed viability (i.e., what proportion of seeds from the accession contained a live embryo in the TTC testing) and post-storage germination rate (i.e., what proportion of embryos from the individual germinated in the ER testing), TTC and ER data for each *Musa* accession were fitted to the NMDS ordination using the vegan ordisurf function, which uses generalised additive models to fit a smooth response surface and is therefore appropriate for a non-linear relationship between the ordination and variable.

The impact of habitat and *Musa* species on the variation in community composition–both for the subset of common taxa visualised in the NMDS and for all OTUs including rare taxa–was tested with permutational multivariate analysis of variance (PERMANOVA) implemented in the vegan adonis and adonis2 functions using Bray-Curtis dissimilarity and 999 permutations. PERMANOVA with adonis considers variables sequentially, meaning that the test is performed on the first variable provided and the residual unexplained variance is left to be explained by the next variable, and so on. As variables can be correlated with each other, the order in which variables are added to the adonis formula impacts the results. In order to determine the unique impact of variables irrespective of order, i.e., marginal effect size (marginal *R*^2^), we used the adonis2 function with the by = “margin” option, which reports the variance that is not explained by any of the other variables. The variables were then tested with adonis in order of decreasing marginal effect size to assess the total effect size (*R*^2^). The vegan betadisper function was also used for permutational analysis of multivariate dispersions (PERMDISP) to assess whether data dispersion was uniform for each variable, as when sample sizes are unbalanced varying data dispersion can result in a significant PERMANOVA test even if group composition is not significantly different (Anderson and Walsh, 2013). The PERMDISP null hypothesis is that there is no difference in dispersion between groups, and so a significant *p*-value indicates that dispersion is not consistent.

In order to determine which of the habitats had significantly different community composition from the others, pairwise PERMANOVA was performed on both the subset of common taxa used in the NMDS as well as all OTUs including rare taxa. This was done using the pairwise.perm.manova function from the R package RVAideMemoire v0.9-78 (Hervé, 2020) with 999 permutations and multiple testing *p*-value correction using the Benjamini–Hochberg method (Benjamini and Hochberg, 1995). Difference in diversity–according to Shannon and Simpson diversity indices, both calculated with the vegan diversity function–and abundance of fungi per *Musa* accession for each habitat was assessed using the TukeyHSD function. All results were plotted in R with the ggplot2 v3.3.0 package (Wickham, 2016). Ellipses for each habitat in the NMDS plot were generated with the stat_ellipse function in ggplot2.

### *Fusarium* Phylogenetic Analysis

Given the abundance of *Fusarium* in our dataset, a genus-specific phylogeny was reconstructed to elucidate the relationships of our *Fusarium* OTUs with already known species. While the UNITE identification described above recovered many 5.8S-partial LSU OTUs to apparent species level, it has been shown that the ITS locus is not sufficiently variable for species delimitation within this particular genus (Geiser et al., 2004). For this reason, all cultures and direct extractions belonging to the genus, as identified with UNITE and confirmed by T-BAS analyses, were selected for additional amplification of the translation elongation factor 1-α (*EF1α*) gene using the primers EF1 and EF2 (O’Donnell et al., 1998). Amplification and sequencing were performed as above, except for PCR cycle conditions, which were informed by da Silva et al. (2014). All new sequences were deposited in GenBank (MW319587-MW319636). OTUs based on *EF1α* sequences were also delimited as above.

Representative sequences for each OTU from this study (as provided by USEARCH) were aligned with already published *EF1α* data and, in addition to *EF1α*, RNA polymerase II largest (*RPB1*), and second largest subunit (*RPB2*) sequences were also taken from the MycoBank website^2^. Taxon sampling was guided by O’Donnell et al. (2013), with the addition of taxa from the *Fusarium oxysporum* species complex (FOSC) (Maryani et al., 2019) and *Fusarium musae* (Van Hove et al., 2011) and *Neonectria coccinea* and *Cylindrocarpon cylindroides* were selected as the outgroup (Supplementary Table 2). Sequences for each gene were aligned using MUSCLE v3.8.425 (Edgar, 2004) and ambiguous regions were manually delimited and removed in AliView v1.17 (Larsson, 2014). Much of the variability in *EF1α* that makes it a valuable marker for *Fusarium* is located across three introns (Geiser et al., 2004), so introns were isolated from protein-coding regions and Gblocks v0.91b (Castresana, 2000) was used to select adequately aligned intron sites, with the “Allow gap positions” option to prevent loss of highly variable sites. To check for topological incongruence between genes, a maximum likelihood (ML) search was performed on individual alignments–partitioned by introns and codon position for protein-coding regions–using the GTRGAMMA substitution model with bootstrapping over 1000 replicates in RAxML v8.2.9 (Stamatakis, 2014). Conflicts between gene trees (defined as ≥70% bootstrap support (BS) for contradictory relationships) were manually identified for each of the three pairwise comparisons with help from the compat.py script (Kauff and Lutzoni, 2002, 2003) run in Python v3.7.9 using Biopython v1.78 (Cock et al., 2009). Taxa responsible for conflicts were removed. The three loci were concatenated using SequenceMatrix (Vaidya et al., 2011) and partitioned by gene, codon position and *EF1α* introns for the ML search, performed as above for individual gene trees–see https://github.com/Rowena-h/MusaEndophytes for the raw alignment and tree files. Species names were checked in Species Fungorum^3^ and the species tree was plotted in R using ggtree v2.3.4 (Yu et al., 2017).

## Results

### Most Endophyte-Colonised *Musa* Seeds Contained a Single OTU

**FIGURE 1:**
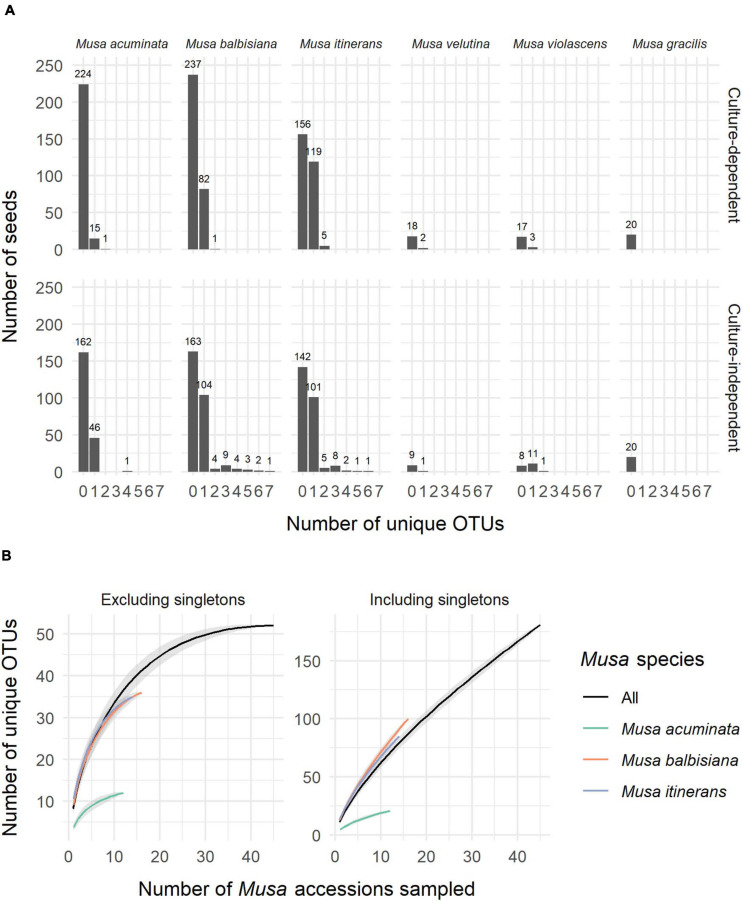
**(A)** The number of unique OTUs per seed for each species of *Musa* from both the culture-dependent and independent approaches. **(B)** Species accumulation curves of OTUs by number of *Musa* accessions sampled, both excluding and including singletons and showing distinction between the three most sampled *Musa* species. Standard error is shaded grey around the lines.

ITS-partial LSU sequences of fungal endophytes were obtained from 533 *Musa* seeds, 31% of the total 1,710 seeds used in this study (+90 control seeds). One fungal isolate per seed was most commonly found, however up to 7 unique OTUs were detected via cloning in a small number of seeds (Figure 1A). Of the most sampled *Musa* species, *M. acuminata* had the lowest number of fungal isolates relative to total seeds while *M. itinerans* had the highest. No fungi were detected in *Musa gracilis*, however only one accession was sampled, which was also the case for *M. violascens* and *M. velutina*.

### *Lasiodiplodia*, *Fusarium*, and *Aspergillus* Were the Most Common Genera

Not including duplicate clones, 642 sequences (GenBank accession numbers MW298868-MW299510) were clustered into 181 OTUs, of which 125 (69%) were singletons. Species accumulation curves including singleton OTUs were almost linear and with a high gradient, while the curves excluding singletons approached an asymptote, indicating that many rarer OTUs remain to be discovered but a considerable proportion of the most common OTUs were captured (Figure 1B).

**FIGURE 2:**
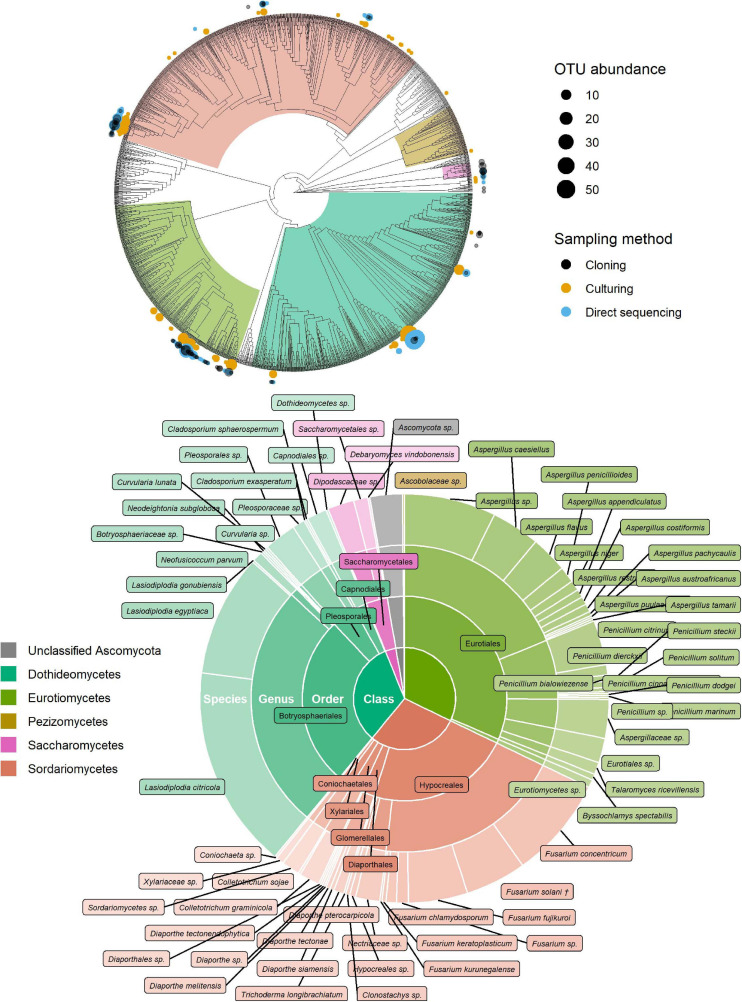
Identification of ascomycete OTUs according to UNITE and phylogenetic placement in the T-BAS Pezizomycotina v2.1 tree. OTUs from this study are indicated on the T-BAS tree by circles on tips (top) with size proportional to number of times the OTU was detected and colour showing sampling method. Taxon classification as agreed by UNITE and T-BAS is summarised in a pie chart (bottom). (†) *Fusarium solani* = *Neocosmospora solani* (Sandoval-Denis et al., 2019).

**FIGURE 3:**
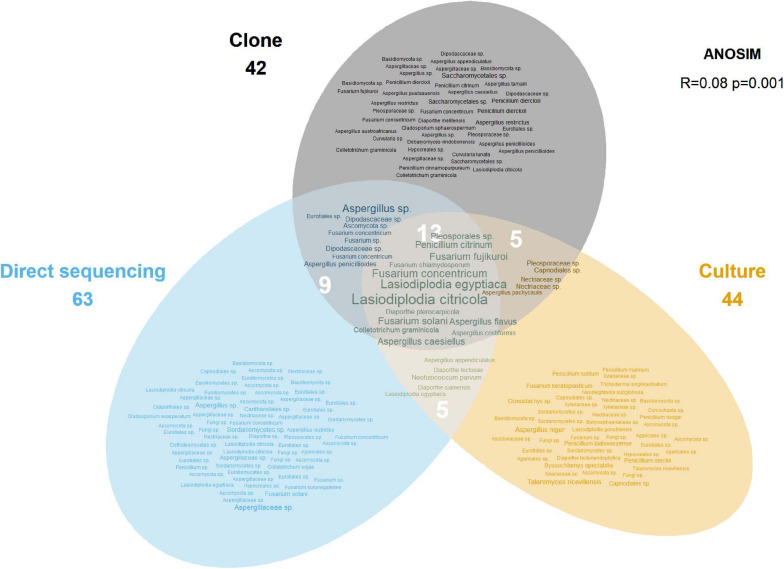
Euler diagram showing the OTUs recovered by each sampling approach. The size of labels is proportional to the number of occurrences for that OTU. Numbers under method labels and in intersections indicate the total number of OTUs for the corresponding approach(es). The ANOSIM result in the top right indicates the statistical significance of the different approaches.

Of the 181 OTUs, UNITE and T-BAS classified the vast majority to the Ascomycota (162, 90%), with a few belonging to the Basidiomycota (12, 7%) and the remaining as unclassified Fungi (7, 4%) (Supplementary Data Sheet 1). In almost equal proportion, most of the ascomycete OTUs fell in the classes Dothideomycetes, Eurotiomycetes, and Sordariomycetes (in order of abundance), in the respective orders of Botryosphaeriales, Eurotiales, and Hypocreales (Figure 2). The three most common genera were *Lasiodiplodia*, *Fusarium*, and *Aspergillus* (with 161, 123, and 117 occurrences, respectively), which together accounted for almost two thirds of the total number of sequenced endophytes. The most abundant OTUs were recovered from all sampling approaches–culture-dependent and culture independent (with additional cloning)–however each approach detected rare OTUs not found by the others (Figure 3). Data dispersion was even across methods (betadisper *p* = 0.33) and ANOSIM indicated that, while communities were significantly different according to different detection methods (*p* = 0.001), the strength of these differences between methods was relatively low (*R* = 0.08). 10 OTUs from inside the seeds were also isolated pre-sterilisation on the outside of seeds (Supplementary Table 3), but as all the surface sterilisation imprint controls showed no fungal growth, we were confident that these OTUs were not contaminants.

### Endophyte Community Composition, Diversity and Abundance Changed With Habitat

**FIGURE 4:**
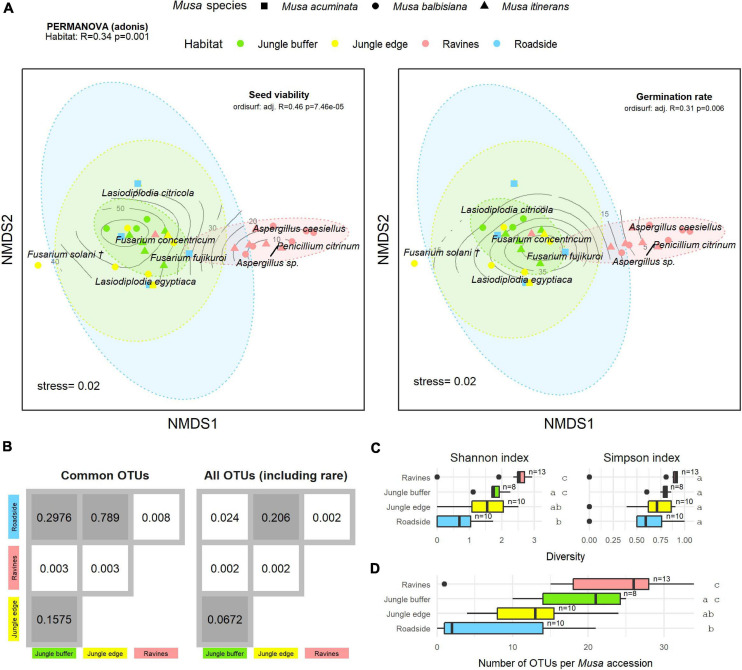
**(A)** Non-metric multidimensional scaling (NMDS) plot of the most common OTUs, produced with metaMDS, fitted with post-storage seed viability (left) and germination rates (right). Contour lines indicate the fit of the seed viability and germination rate variables to the ordination using the ordisurf function, showing which points are associated with higher or lower seed viability. Each point represents one *Musa* accession, with shape showing host *Musa* species and colour showing habitat, while OTUs are shown in italic text. (†) *Fusarium solani* = *Neocosmospora solani* (Sandoval-Denis et al., 2019). Ellipses were generated with the stat_ellipse function in ggplot2. The PERMANOVA result in the top left indicates significant difference in endophyte community composition between habitats. **(B)** Matrix of pairwise PERMANOVA *p*-values showing whether endophyte community was significantly different between pairs of habitats, both for the subset of common OTUs visualised in the NMDS and including rare OTUs. Grey boxes indicate non-significant *p*-values (>0.5). Diversity according to Shannon and Simpson indices **(C)** and abundance of OTUs **(D)** per *Musa* accession in each habitat. Groups with significant difference of means as calculated by TukeyHSD are shown by letters on the right of the plots. Sample size (number of accessions) is shown to the right of boxes.

There was a significant difference in endophyte communities across habitats when considering the most common OTUs (pooled from all detection methods) from *M. acuminata*, *M. balbisiana*, and *M. itinerans* accessions (adonis2 marginal *R*^2^ = 0.32, *p* = 0.001; adonis *R*^2^ = 0.34, *p* = 0.001) and also when including rare taxa, although with a smaller effect size (adonis2 marginal *R*^2^ = 0.18, *p* = 0.001; adonis *R*^2^ = 0.21, *p* = 0.001). *Musa* species was not found to be a significant factor for variance of taxa (Table 1). PERMDISP found data dispersion of common taxa to be similar across *Musa* species but not across habitats: dispersion was greatest in the habitat with the smallest sample size (roadside), suggesting a liberal PERMANOVA bias (Supplementary Figure 2) (Anderson and Walsh, 2013). However, PERMANOVA, PERMDISP, and NMDS together suggested that habitat was associated with both location and dispersion of the data. The NMDS visualisation showed that the ellipses for the jungle buffer, jungle edge and roadside habitats overlapped, but with data dispersion increasing with level of habitat disruption: from jungle buffer (least disrupted, most tightly clustered) to roadside (most disrupted, least tightly clustered) (Figure 4A). The pairwise PERMANOVA analysis confirmed that these three habitats were not significantly different to each other in community composition for the common taxa visualised in the NMDS, while they were all significantly different from the ravine habitat (Figure 4B), which formed a separate cluster in the NMDS (Figure 4A). When including rare OTUs in the pairwise PERMANOVA, however, community composition was also significantly different between jungle buffer and roadside habitats (Figure 4B). Both diversity and abundance of endophytes per accession showed the same trend across habitats, with greatest diversity and abundance in the ravine habitat and least in the roadside habitat, with TukeyHSD identifying three statistically distinct groups for both Shannon diversity and abundance, although Simpson diversity was not statistically significant between habitats (Figures 4C,D). Oil palm plantation accessions and the RBG, Kew accession were excluded from the main analyses due to low sample size (and as the latter was a geographical outlier), but endophyte abundance was comparatively low for both habitats (Supplementary Figure 3).

**TABLE 1:** Results of the statistical tests on Bray-Curtis dissimilarity matrices of both the subset of common OTUs visualised in the NMDS and all taxa including rare OTUs.

		**PERMANOVA**				**PERMDISP**
		**adonis2**		**adonis**		**Betadisper**
**Dataset**	**Variable**	**Marginal *R*^2^**	***p***	***R*^2^**	***p***	***p***
Common taxa	Habitat	0.32	0.001	0.34	0.001	0.0310
	*Musa* species	0.07	0.055	0.07	0.055	0.0987
All taxa	Habitat	0.18	0.001	0.21	0.001	0.0059
	*Musa* species	0.08	0.101	0.08	0.101	1.25E-07

Fitting post-storage seed viability (TTC) to the NMDS ordination (ordisurf adjusted *R*^2^ = 0.46, *p* = 7.46e-05) showed seed viability to have a non-linear relationship with the community structure, with accessions in the ravine habitat cluster and *Penicillium* and *Aspergillus* OTUs associated with lower viability measures and accessions in the jungle buffer habitat associated with higher viability measures (Figure 4A). Germination rate (ER) showed a similar relationship (ordisurf adjusted *R*^2^ = 0.31, *p* = 0.006).

### *Fusarium* Strains Were Phylogenetically Resolved to the *Fusarium fujikuroi*, “*Fusarium” solani*, and *Fusarium incarnatum-equiseti* Species Complexes

**FIGURE 5:**
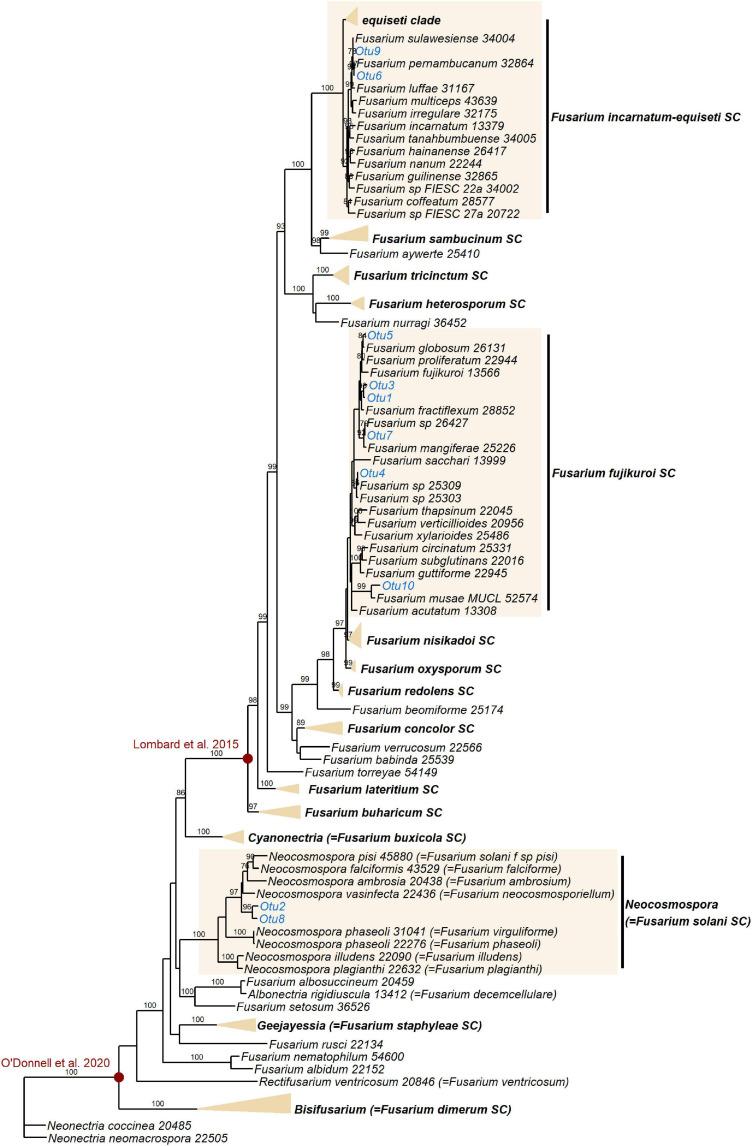
Maximum likelihood RAxML tree reconstructing relationships of 130 taxa of *Fusarium* and closely related genera, including the *EF1α* OTUs delimited in this study (indicated in blue). Bootstrap support values ≥ 70% are shown on internodes. Genera and *Fusarium* species complexes (SC) which weren’t represented by any OTUs in this study are collapsed where possible–the yellow triangles are vertically scaled for ease of visualisation, with horizontal length representing the longest branch in the species complex. Species complex and genus names are shown in bold. Red circles indicate generic limits of *Fusarium* proposed by Lombard et al. (2015) and O’Donnell et al. (2020).

Additional *EF1α* sequencing and OTU clustering of the *Fusarium* taxa produced 10 *EF1α* OTUs. Phylogenetic analysis resolved these in the *incarnatum* clade of the *Fusarium incarnatum-equiseti* species complex (FIESC), in the *Fusarium solani* species complex (FSSC)–which has recently been reassigned to the genus *Neocosmospora* (Sandoval-Denis et al., 2019)–and in the *Fusarium fujikuroi* species complex (FFSC), with most OTUs placed within the latter (Figure 5). Disregarding the naming of taxa, our phylogeny was in general agreement with the most comprehensive phylogenies of the genus (O’Donnell et al., 2013, 2020), with the exception of not recovering geographically grouped clades (Asian, African, and American) in the FFSC, which was also one of the only species complexes that was not significantly supported. Across the whole phylogeny, 68% of all internodes were significantly supported. Extremely short branch and internode lengths indicated rapid divergence in the FFSC and FIESC clades, as well as in other species complexes such as FOSC and *Fusarium redolens* species complex.

## Discussion

In this study, we used both a culture-dependent and culture-independent approach to assess the diversity of endophytes in stored banana CWR seeds. In an example of the value of collections, we demonstrated the feasibility of endophyte discovery from seed banks, many strains of which can be isolated in culture for future study. By using cloning versus next-generation-sequencing methods for the culture-independent detection of seed endophytes, we were able to economically sequence individual seeds (rather than a pooled sample) to determine the endophyte capacity of the *Musa* seeds from the culture-independent approach, which could then be combined with the data on number of endophytes isolated in culture per seed. Of the seeds containing endophytes, the number of unique OTUs was biased toward one for both sampling approaches (Figure 1A), which suggests that there is some level of competitive exclusion in the limited physical space of the seed, as posited by Raghavendra et al. (2013). This is also in agreement with recent work on seeds from various alpine plants, which showed that, while bacterial endophytes appear to interact positively, fungi are usually mutually exclusive (Wassermann et al., 2019). Similarly, during pathogenic invasion of radish seeds, it was found that a fungus altered the fungal endophyte community while a bacterium had no effect on either bacterial or fungal endophytes, although the authors noted that the different infection routes and thus microhabitats of the two pathogens could have contributed to the observed community differences (Rezki et al., 2016). Our seeds were all pre-dispersal (as all MSB seeds are), so there is also the possibility that the endophyte capacity was influenced by the lack of opportunity for seeds to acquire fungi from the soil, which is known to be a source of much seed endophyte diversity (e.g., U’Ren et al., 2009; Sarmiento et al., 2017). More insight into the dynamics of endophyte seed colonisation is needed, and would benefit from experimental inoculation combined with *in situ* visualisation of the physical space endophytes inhabit within the seed (e.g., Rath et al., 2014; Vági et al., 2014). Previous work on the specific localisation of seed endophytes has established that it varies depending on the species in question: some endophytic species are known to only be found in the seed coat (Oldrup et al., 2010) while others such as grass symbionts are found in the embryo and endosperm (Philipson and Christey, 1986; Zhang et al., 2017). Although in this study we did not establish the exact localisation of endophytes within the *Musa* seeds, the fact that we both cultured and directly sequenced many fungi from whole seeds whereas ER testing showed no or minimal “contamination” (i.e., any fungal growth from extracted embryos) suggests that most of the OTUs may have been located outside the embryo (Supplementary Table 1). However, as the ER testing only applies to culturable fungi and the embryo may contain endophytes that can only be detected through direct sequencing (Figure 3), with this data we cannot conclusively comment on the localisation of our taxa within the seeds. We also checked for OTUs present on the seed surface (Supplementary Table 3)–we were confident that these OTUs were also found as endophytes inside the seeds and not contaminations as we performed culture imprint controls to confirm the efficacy of the surface sterilisation method. Being both inside and outside the seed indicates that these strains were more likely generalists, horizontally transferred, for instance, from fruit to seed, rather than vertically transmitted endophytes, which would not be expected to be found outside the seed as well.

The genera found in the *Musa* seeds were largely similar to previous studies of *Musa* endophytes from roots and leaves (Sikora et al., 2008; Wang et al., 2014; Zakaria et al., 2016; Zakaria and Aziz, 2018), as well as other tropical tree endophytes, such as from cacao branches (Rubini et al., 2005) and rubber leaves (Vaz et al., 2018) and tropical orchid roots (Bayman and Otero, 2007). The most commonly found genera, *Lasiodiplodia*, *Fusarium*, and *Aspergillus*, are all ubiquitous in both endophytic and other contexts. The genus *Lasiodiplodia* is best known for the species *Lasiodiplodia theobromae*, a prevalent endophyte in the global tropics (Salvatore and Andolfi, 2020), but also an infamous pathogen of tropical fruit trees. For instance, *L. theobromae* has been found to cause crown rot in commercial banana (Sangeetha et al., 2012) and–among other *Lasiodiplodia* strains–stem and fruit rot in papaya (Netto et al., 2014) and dieback in mango (Rodríguez-Gálvez et al., 2017). Goos et al. (1961) similarly found *L. theobromae* (using the synonym *Botryodiplodia theobromae*) to be pervasive in seeds of *Musa* spp., although they did not report whether the colonised seeds or resulting plants had disease symptoms. They also found *L. theobromae* exclusively in the seed coat and micropylar plug versus the endosperm or embryo and echoed our above hypothesis that it is transferred from fruit to seed. This was also supported in *Musa ornata*, for which *Lasiodiplodia* colonisation was observed in all cases apart from those where embryos were removed from seeds under aseptic conditions (Burgos-Hernández et al., 2014). The two prevalent *Lasiodiplodia* OTUs in this study were classified as *Lasiodiplodia citricola* and *Lasiodiplodia egyptica*, both of which were first described from diseased plants: *Citrus* spp. showing “branch dieback, cankers and fruit rot” (Abdollahzadeh et al., 2010) and mango suffering dieback (Ismail et al., 2012), respectively. *L. egyptica* has also been implicated in stem-end rot of coconut (Rosado et al., 2016) and *L. citricola* in disease of English walnut (Chen et al., 2013). Although we could find no reports of these species as endophytes, their relatively recent description makes it likely that their full extent of occurrence has not been revealed. Sequencing phylogenetically informative loci for these endophytic *Lasiodiplodia* strains–e.g., *EF1α* and TUB2 (de Silva et al., 2019)–will be desirable in the future to confirm their identity with phylogenetic analysis.

Like *Lasiodiplodia*, multiple *Fusarium* strains are phytopathogenic (Aoki et al., 2014), and *Fusarium oxysporum* and *Fusarium graminearum* both feature in the top 10 most economically/scientifically important fungal plant pathogens (Dean et al., 2012). This is certainly relevant to commercial banana crops, which are under threat from Foc TR4, the causal agent of Fusarium Wilt (Dita et al., 2018). Fungi in the genus *Fusarium* are also known to be common endophytes in *Musa* species, however, having been previously isolated from either wild or commercial *Musa* in China, Thailand and Guatemala (zum Felde et al., 2003; Sikora et al., 2008; Wang et al., 2019). The species complexes represented in this study–FIESC, FFSC, and FSSC–are all known to comprise both phytopathogens and endophytes (Kavroulakis et al., 2007; Aoki et al., 2014; Niehaus et al., 2016; Bilal et al., 2018; Wang et al., 2019), and additionally both the FIESC and FSSC contain species that act as opportunistic human pathogens (Zhang et al., 2006; O’Donnell et al., 2009). Even within species, *Fusarium* strains can differ greatly in their proclivity to cause disease in their plant host–*in vitro* expression of secondary metabolites (including phytohormones and mycotoxins) in an orchid endophytic *Fusarium proliferatum* strain was shown to be distinct from expression in a pathogenic *F. proliferatum* strain (Niehaus et al., 2016). It has also been demonstrated that commercial banana roots can be protected from nematodes by endophytic FOSC strains (zum Felde et al., 2003; Mendoza and Sikora, 2009), the same species complex to which Foc TR4 belongs. We should highlight that the taxonomy of *Fusarium* is highly contested (Summerell, 2019). Recent dismantling and splitting of certain *Fusarium* species complexes into several distinct genera (Lombard et al., 2015), including reassigning species in the FSSC to the genus *Neocosmospora* (Sandoval-Denis et al., 2019), has received pushback, the main opposing argument being that a broader generic concept benefits practitioners dealing with human and plant pathogens (O’Donnell et al., 2020). Different perspectives on the limits of the generic concept of *Fusarium* (illustrated in Figure 5) will no doubt continue to be debated.

Unlike the former two genera, *Aspergillus* is not known predominantly for plant-associated taxa, but rather for globally distributed air and soilborne saprotrophs, with some species infamously acting as opportunistic human pathogens (Bennett, 2010; Latgé and Chamilos, 2020). Nonetheless, *Aspergillus* species are also frequently found as endophytes, and an endophytic *Aspergillus fumigatus* strain isolated from *Oxalis corniculata* roots has been shown to promote growth in rice (Bilal et al., 2018). Intriguingly, the most prevalent OTU for the genus in this study was classified as *Aspergillus caesiellus*, which has been reported as a marine endophyte of seagrasses and sponges (Liu et al., 2010; Subrmaniyan et al., 2018). The second most prevalent OTU was *Aspergillus flavus*, a ubiquitous soil fungus known for contaminating stored grains with aflatoxins, and also an agent of aforementioned opportunistic diseases in animals and humans (Amaike and Keller, 2011). The range of plant-fungal interactions that are observed in these three genera emphasises the ongoing question we face for the endophytic lifestyle as a whole–how can we distinguish mutualistic or commensal endophytes from latent pathogens? Greater exploration of the genomic features and expression profiles of endophytes is required to tackle this issue, and seed banks provide an excellent resource for targeting economically, environmentally and scientifically important plant hosts from which to isolate strains for this purpose.

A relatively modest sampling effort was required to isolate the majority of common OTUs found across *Musa* species (Figure 1B), in agreement with other microfungi community studies (e.g., Paulus et al., 2006; Tisthammer et al., 2016; Vaz et al., 2018), but the vast majority of OTUs were singleton or low-abundance, a known phenomenon in microbial diversity (Lynch and Neufeld, 2015; Jia et al., 2018). A disproportionate number of rare taxa can obstruct community composition visualisation methods such as NMDS, and so low abundance taxa are often filtered out in order to visualise structural patterns (e.g., Miller et al., 2016; Huang et al., 2018; U’Ren et al., 2019). This is distinct from the practise of removing rare/singleton OTUs from high throughput sequencing datasets in case of sequencing artefacts (e.g., Brown et al., 2015). Poos and Jackson (2012) discussed two arguments for removal of rare taxa in multivariate analysis in the context of bioassessments: statistical impact (“rare species provide limited interpretative value and add noise”) and biological impact (“rare taxa do not provide meaningful information beyond that captured by more common species”). For our comparison of endophyte communities between different host habitats, PERMANOVA analysis found the effect size of habitat on community variance to be greater when excluding rare taxa (Table 1), but when comparing the significance of individual habitats with pairwise PERMANOVA the inclusion of rare taxa revealed differences between habitats that were not found from the common taxa alone (Figure 4B). This challenges the “biological impact” argument above, however removal of rare taxa remains a practical compromise to enable visualisation of at least a subset of the community structure. A valid question is whether the rare taxa that were detected are conditionally rare (i.e., their abundance is based on abiotic conditions), or permanently rare. We would need repeated samples over time to clarify this, and although outside the scope of this study, seed bank collections are excellently positioned for addressing this in the future.

While the impact of “edge effect”–change in community structure at the boundary of habitats, whether natural or from e.g., encroaching human land use or bisecting roads–has been well documented for plant communities (Skole and Tucker, 1993; Harper et al., 2005; Kunert et al., 2015), it is far less studied in fungi (Crockatt, 2012; Ruete et al., 2016), and, to our knowledge, the concept has not been addressed in the context of endophytes. Our results comparing the jungle buffer, jungle edge and roadside habitats suggest that there may indeed be some level of edge effect manifested in the seed mycobiome of these *Musa* accessions, both in diversity and abundance (Figures 4C,D). While community composition did not differ between these three habitats for the most common taxa, when including rare taxa there was a significant difference between the jungle buffer and roadside habitats (Figure 4B). Seeing a community difference between these habitats when including rare and not just common taxa suggests that the rare endophytes may be more sensitive to edge effects, which would be consistent with the concept of biotic homogenisation as a result of ecosystem disruption (Mckinney and Lockwood, 1999; Parra-Sanchez and Banks-Leite, 2020). This is also supported by the fact that the Shannon index, which is sensitive to rare species, found a significant difference in diversity, while the Simpson index, which is sensitive to abundant species, did not (Morris et al., 2014). These results come with the caveat that the habitats as defined in this study are interpreted from the MSB seed collection data, which were not recorded with any particular study design in mind, and as such some entries are more complete than others and there can be subjectivity in how to infer habitat from the collection notes. While the extensive metadata attached to natural history collections can be incredibly powerful for studying patterns of biodiversity (Andrew et al., 2018, 2019; Funk, 2018; Pearce et al., 2020), the application of that data must be done with care.

Fitting post-storage seed viability (TTC) and germination rate (ER) data to the NMDS visualisation showed jungle habitat accessions to be associated with highest seed viability and ravine habitat accessions to be associated with lowest seed viability. As these assessments specifically measured post-storage viability/germination, we relied on the assumption that the same collection standards and procedures were always adhered to, as other factors have been shown to impact *Musa* seed viability such as maturity of the seed at collection and the speed of drying before cold storage (Kallow et al., 2020). Nonetheless, these results highlighted *Fusarium* and *Lasiodiplodia* strains, which would be particularly interesting to trial in experimental inoculation studies, to verify whether they impact the survivability of *Musa* seeds in storage, or indeed the germination rates of fresh seeds. Endophytic *Fusarium* strains have previously been found to promote germination and seedling growth of an Indonesian peatland grass (Tamura et al., 2008) and germination of orchid seeds (Bayman and Otero, 2007). In addition to the aforementioned roles of *L. theobromae* in tropical fruit tree diseases, it has also been implicated in seed rot, for instance of slash pine (Cilliers et al., 1993), and to cause reduced germination rates in aridan and coconut seeds (Dugan et al., 2016). The role of seedborne *L. theobromae* on germination may be more nuanced, however, as it has been found to produce fatty acid esters, which can alternately inhibit and promote tobacco seed germination and seedling growth (Uranga et al., 2016). Considering the pathogenic role of numerous *Lasiodiplodia* species discussed above, it is interesting that this study saw *Lasiodiplodia* strains to be prevalent in *Musa* accessions with comparatively high post-storage seed viability. A previous study of *in vitro* germination of both stored and fresh *M. ornata* seeds found *Lasiodiplodia* to persistently infect seeds, with the implication that these seeds then decayed (Burgos-Hernández et al., 2014). Goos et al. (1961) reported a similar result for seeds of various *Musa* spp. in aseptic conditions, however they noted that germination was not significantly affected by *Lasiodiplodia* colonisation under “greenhouse conditions.” This raises the question as to whether the pathogenic potential of *Lasiodiplodia* strains in *Musa* seeds is influenced by the abiotic conditions and/or co-occurrence of other fungi. Of course, without isolating specific strains and performing controlled pathogenicity tests, it is impossible to answer this, as different fungal strains can vary in their ability to cause disease regardless of secondary factors such as environment. It would also be interesting to look at the endosymbiotic or “endohyphal” bacteria associated with our strains, as these have been found, in rare cases, to be capable of effecting (pre-storage) seed germination and viability in a neotropical tree (Shaffer et al., 2018).

An interesting result was that the abundance of endophytes per *Musa* accession was greatest in the ravine habitat (Figure 4B), the same habitat that was adversely correlated with post-storage seed viability. Returning to the ambiguity of the endophytic lifestyle, this again raises the issue that it is not the mere presence of endophytes, but the identity of specific strains that may have implications for stored seeds. The difference in abundance and community composition in the ravine habitats could partially be explained by altitude, although unfortunately there was not sufficient altitude data for all accessions in the MSB records to test this. Previous studies on the effect of altitude on endophyte communities have suggested an inconsistent relationship (Granath et al., 2007; Hashizume et al., 2008; Zubek et al., 2009; Bonfim et al., 2016), no doubt partially due to the large number of confounding factors associated with changing altitude, such as variation in the host plant assemblages, as host availability is believed to be a main driver of endophyte community composition (U’Ren et al., 2019). Host availability may also have been a key factor as to why accessions in oil palm plantations and a botanical garden had low endophyte abundance (Supplementary Figure 3). Although the sample size for these habitats was too small to include them in the main analyses, these were the only managed habitats with, presumably, the least natural co-occurring plant assemblages.

There are a number of considerations for seed banking in the context of endophytes that are important to raise for future discussion and research. Firstly, our results show that habitat of the host plants from which seeds are collected could impact the associated endophyte communities, which may potentially have downstream consequences for seed survival. Collecting seeds from individuals in a range of habitats with different co-occurring plant species may be advisable to conserve endophytic diversity. As current seed bank protocol is to collect seeds pre-dispersal, before horizontal transmission of fungi from soil to seed, what, if any, impact does this have on subsequent viability of the seeds or health of the descendent plants? To our knowledge, only one study has made a direct comparison of endophytic communities in pre and post-dispersal seeds for the same plant individual, finding fewer endophytes in pre-dispersal seeds of a neotropical tree species, none of which were successfully isolated in culture (Gallery et al., 2007). Studies of buried seeds have shown that seeds acquire diverse endophytes through horizontal transmission from the soil (e.g., U’Ren et al., 2009; Sarmiento et al., 2017), but are also vulnerable to soilborne pathogens (Gallery et al., 2010). It could then be that the current protocol of storing pre-dispersal seeds is preferable, as it limits the acquisition of potential pathogens while still allowing the possibility for mutualistic endophytes to be vertically transmitted from the parent plant. The dynamics of endophyte transmission are likely to be highly variable between different plant groups, however, and more studies of seeds from different hosts, geographical areas and dispersal stages are needed to identify the optimal collection procedure for healthy microbiomes of stored seeds.

## Conclusion

This study has demonstrated that seed banks provide huge potential for research into fungal endophyte communities. As well as being an untapped resource for new fungal diversity, the ability to isolate live strains from almost 40,000 global plant taxa curated by the MSB–a third of which are identified as having significant natural capital value (Liu et al., 2018)–provides far-reaching opportunities for future study of the role of endophytes in plant health. For this reason, although originally designed for conservation of plant genetic diversity, seed banks may have an equally important role in conserving the seed microbiome, and much more discussion and research is needed on how the seed collection and storage procedure can best accommodate this.

## Data Availability Statement

The datasets presented in this study can be found in online repositories. The names of the repository/repositories and accession number(s) can be found in the article/Supplementary Material.

## Author Contributions

SK and JD provided the samples and contributed to the writing of the manuscript. RH, TL, ED, JO, and CM carried out the molecular data collection. BP performed the embryo rescue testing. RH and EG designed and implemented the analysis of the results and wrote the manuscript. EG conceived the original idea and supervised the project. All authors contributed to the article and approved the submitted version.

## Conflict of Interest

The authors declare that the research was conducted in the absence of any commercial or financial relationships that could be construed as a potential conflict of interest.

## Footnotes

1 www.promusa.org

2 https://fusarium.mycobank.org/

3 http://www.speciesfungorum.org/

## Supplementary Material

Supplementary Figure 1 Scree plot up to 10 dimensions for the NMDS analysis (top) and stress plot for the chosen number of 6 dimensions (bottom).

Supplementary Figure 2 Dispersion of data for each habitat for both the common taxa used in the NMDS and all taxa including rare OTUs, as assessed with betadisper. Sample size (number of accessions) is shown to the right of the plots. Sample size is less for the roadside habitat in the “All OTUs” category than in the diversity and abundance analyses (Figures 4C,D) because accessions with no OTUs detected are removed by betadisper.

Supplementary Figure 3 Abundance of OTUs per *Musa* accession for each habitat including oil palm plantation and botanical garden. Groups with significant difference of means as calculated by TukeyHSD are shown by letters on the right of the plots. Sample size (number of accessions) is shown to the right of boxes.

Supplementary Table 1 Millennium Seed Bank (MSB) serial numbers and metadata associated for the 45 wild *Musa* accessions used in this study.

Supplementary Table 2 GenBank accession numbers for taxa used in the phylogenetic analysis. Accessions in bold were sequenced in this study.

Supplementary Table 3 Summary of OTUs which were also found on the unsterilised seed surface.

Supplementary Data Sheet 1 Classification of OTUs by UNITE and T-BAS and the inferred consensus.
