# Supplementary figures and images for: Seed Banks as Incidental Fungi Banks: Fungal Endophyte Diversity in Stored Seeds of Banana Wild Relatives
Source: Front Microbiol. 2021 Mar 22;12:643731. doi: 10.3389/fmicb.2021.643731 (PMC8024981; doi:10.3389/fmicb.2021.643731)

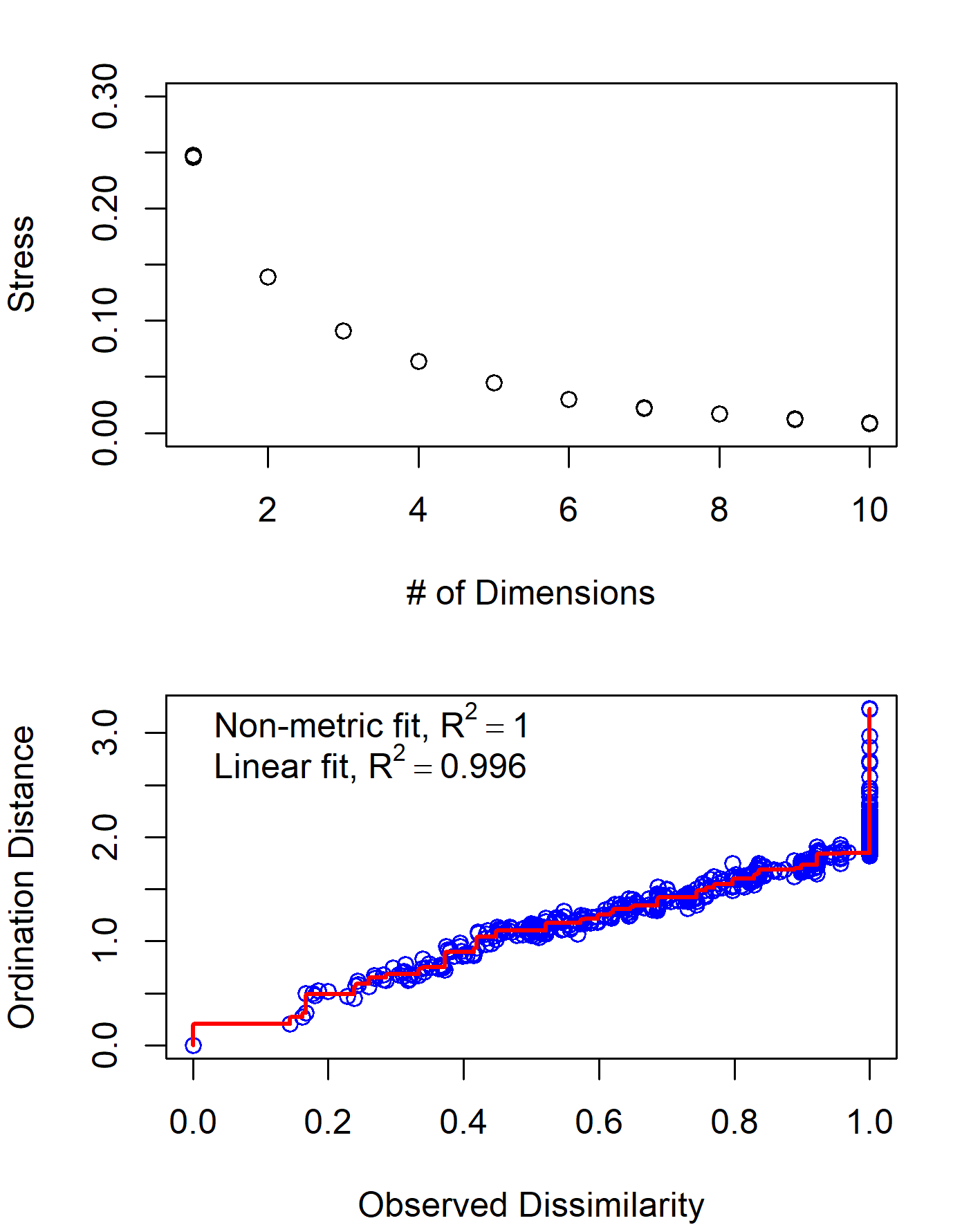

Supplement: Supplementary Figure 1 — Scree plot up to 10 dimensions for the NMDS analysis (top) and stress plot for the chosen number of 6 dimensions (bottom). [file Image_1.tiff]

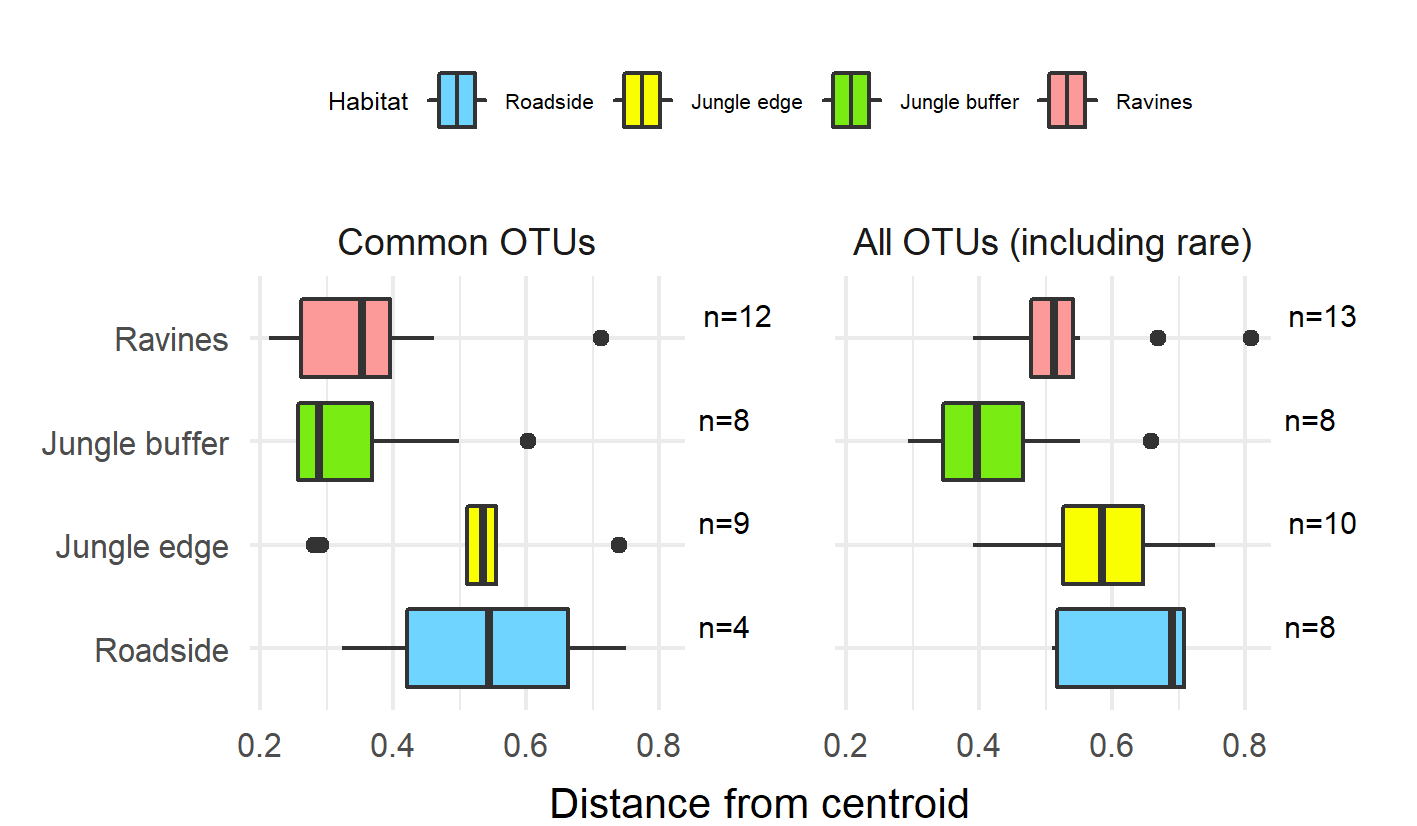

Supplement: Supplementary Figure 2 — Dispersion of data for each habitat for both the common taxa used in the NMDS and all taxa including rare OTUs, as assessed with betadisper. Sample size (number of accessions) is shown to the right of the plots. Sample size is less for the roadside habitat in the “All OTUs” category than in the diversity and abundance analyses (Figures 4C,D) because accessions with no OTUs detected are removed by betadisper. [file Image_2.tiff]

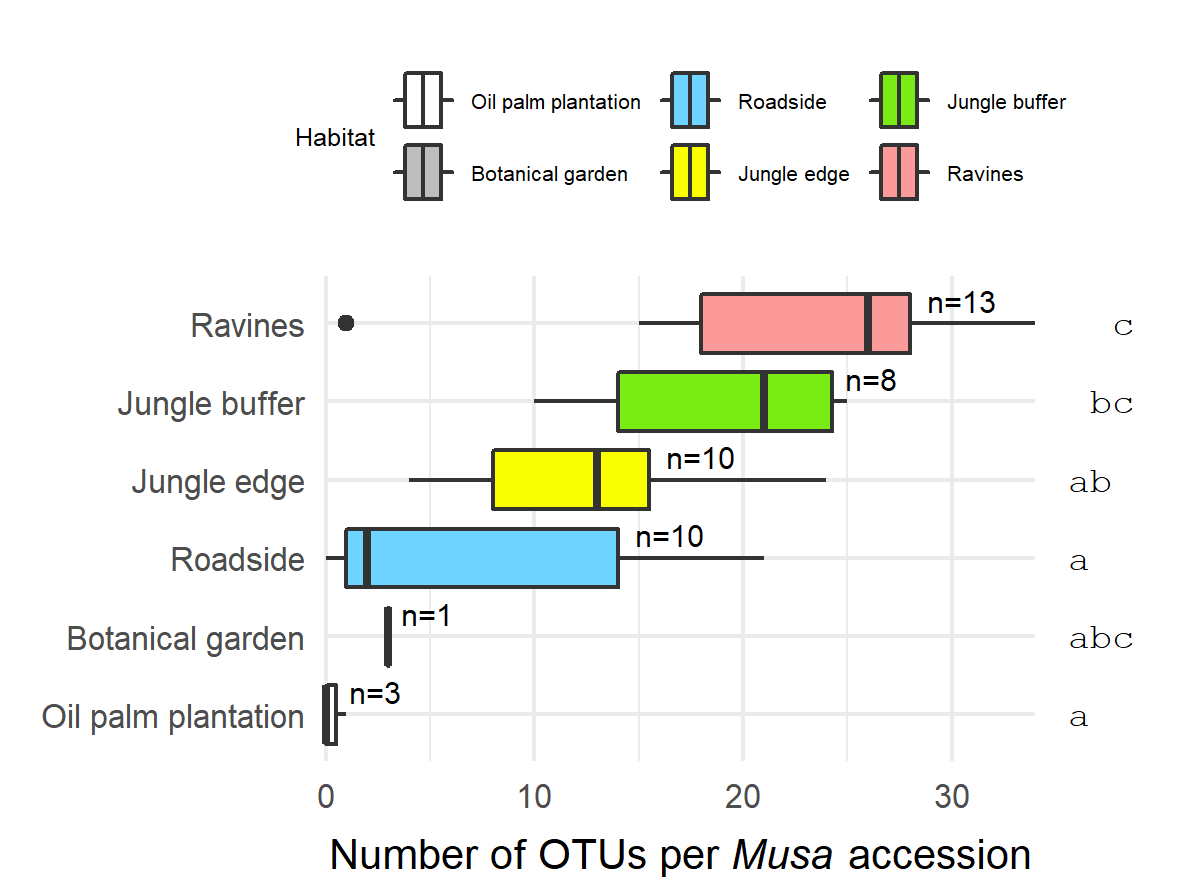

Supplement: Supplementary Figure 3 — Abundance of OTUs per Musa accession for each habitat including oil palm plantation and botanical garden. Groups with significant difference of means as calculated by TukeyHSD are shown by letters on the right of the plots. Sample size (number of accessions) is shown to the right of boxes. [file Image_3.tiff]
