# Supplementary material for: Seed Banks as Incidental Fungi Banks: Fungal Endophyte Diversity in Stored Seeds of Banana Wild Relatives
Source: Front Microbiol. 2021 Mar 22;12:643731. doi: 10.3389/fmicb.2021.643731 (PMC8024981; doi:10.3389/fmicb.2021.643731)
Supplement: Supplementary Table 1 — Millennium Seed Bank (MSB) serial numbers and metadata associated for the 45 wild Musa accessions used in this study. [file Table_1.docx]

**Supplementary Table 1**. Millennium Seed Bank (MSB) serial numbers and metadata associated for the 45 wild *Musa* accessions used in this study.

| Serial number | Species | Collection year | Collection location | Seed viability (TTC) (%) | Germination rate (ER) (%) | Contamination (ER) (%) | Habitat |
| --- | --- | --- | --- | --- | --- | --- | --- |
| 836375 | *Musa balbisiana* | 2014 | Vietnam:Ha Tinh Province:Ky Anh District | 56 | 25 | 0 | Jungle buffer |
| 836445 | *Musa itinerans* | 2014 | Vietnam:Ha Tinh Province:Huong Son District | 0 | NA | 0 | Jungle buffer |
| 836467 | *Musa itinerans* | 2014 | Vietnam:Nghe An Province:Thanh Chuong District | 63 | 63 | 0 | Jungle buffer |
| 836478 | *Musa balbisiana* | 2014 | Vietnam:Nghe An Province:Thanh Chuong District | 88 | 10 | 0 | Jungle buffer |
| 836489 | *Musa balbisiana* | 2014 | Vietnam:Nghe An Province:Thanh Chuong District | 86 | 70 | 0 | Jungle buffer |
| 836490 | *Musa itinerans* | 2014 | Vietnam:Nghe An Province:Thanh Chuong District | 52 | 70 | 0 | Jungle buffer |
| 836504 | *Musa itinerans* | 2014 | Vietnam:Nghe An Province:Thanh Chuong District | 88 | 0 | 0 | Jungle buffer |
| 836515 | *Musa itinerans* | 2014 | Vietnam:Nghe An Province:Con Cuong District | 60 | 80 | 0 | Jungle buffer |
| 880079 | *Musa balbisiana* var*. bakeri* | 2015 | Vietnam:Lào Cai Province:Bao Thang District | 6 | 0 | 0 | Ravines |
| 880116 | *Musa itinerans* | 2015 | Vietnam:Lào Cai Province:Sa Pa | 0 | 10 | 10 | Ravines |
| 880127 | *Musa balbisiana* | 2015 | Vietnam:Lào Cai Province:Sa Pa | 0 | 0 | 10 | Ravines |
| 880138 | *Musa itinerans* | 2015 | Vietnam:Lào Cai Province:Sa Pa | 0 | 0 | 10 | Ravines |
| 880149 | *Musa itinerans* | 2015 | Vietnam:Lào Cai Province:Sa Pa | 0 | 0 | 0 | Ravines |
| 880161 | *Musa balbisiana* | 2015 | Vietnam:Lào Cai Province:Sa Pa | 0 | 10 | 0 | Ravines |
| 880172 | *Musa balbisiana* | 2015 | Vietnam:Lào Cai Province:Sa Pa | 9 | 0 | 0 | Ravines |
| 880264 | *Musa itinerans* | 2015 | Vietnam:Lai Châu Province:Tam Ðuong District | 4 | 0 | 0 | Ravines |
| 880323 | *Musa itinerans* | 2015 | Vietnam:Lào Cai Province:Sa Pa | 3 | 0 | 0 | Ravines |
| 880334 | *Musa itinerans* | 2015 | Vietnam:Lào Cai Province:Sa Pa | 0 | 0 | 0 | Ravines |
| 880345 | *Musa balbisiana* var*. bakeri* | 2015 | Vietnam:Lào Cai Province:Sa Pa | 54 | 0 | 0 | Ravines |
| 880356 | *Musa balbisiana* | 2015 | Vietnam:Lào Cai Province:Sa Pa | 0 | 0 | 0 | Ravines |
| 880367 | *Musa balbisiana* | 2015 | Vietnam:Lào Cai Province:Sa Pa | 6 | 0 | 0 | Ravines |
| 880585 | *Musa balbisiana* var*. bakeri* | 2015 | Vietnam:Lai Châu Province:Phong Tho District | 12 | 75 | 0 | Jungle edge |
| 880600 | *Musa balbisiana* var*. bakeri* | 2015 | Vietnam:Lai Châu Province:Phong Tho District | 60 | 80 | 0 | Jungle edge |
| 880622 | *Musa itinerans* | 2015 | Vietnam:Lai Châu Province:Phong Tho District | 23 | 30 | 0 | Jungle edge |
| 880633 | *Musa itinerans* | 2015 | Vietnam:Lai Châu Province:Phong Tho District | 45 | 78 | 0 | Jungle edge |
| 880644 | *Musa itinerans* | 2015 | Vietnam:Lai Châu Province:Phong Tho District | 51 | 30 | 0 | Jungle edge |
| 882671 | *Musa acuminata* | 2015 | Malaysia:Peninsula Malaysia:Pahang | 36 | 0 | 0 | Roadside |
| 882730 | *Musa acuminata* | 2015 | Malaysia:Peninsula Malaysia:Pahang | 46 | 0 | 0 | Roadside |
| 882741 | *Musa acuminata* subsp. *malaccensis* | 2015 | Malaysia:Peninsula Malaysia:Pahang | 83 | 70 | 0 | Roadside |
| 882785 | *Musa acuminata* | 2015 | Malaysia:Peninsula Malaysia:Negeri Sembilan | 48 | 56 | 0 | Roadside |
| 882800 | *Musa acuminata* subsp*. acuminata* | 2015 | Malaysia:Peninsula Malaysia:Negeri Sembilan | 64 | 33 | 0 | Roadside |
| 882811 | *Musa acuminata* subsp*. acuminata* | 2015 | Malaysia:Peninsula Malaysia:Negeri Sembilan | 71 | 0 | 0 | Roadside |
| 882833 | *Musa acuminata* subsp*. acuminata* | 2015 | Malaysia: Peninsula Malaysia: Selangor | 73 | 20 | 0 | Roadside |
| 882877 | *Musa gracilis* | 2015 | Vietnam:Lào Cai Province:Sa Pa | 0 | 0 | 0 | Roadside |
| 882888 | *Musa acuminata* subsp*. malaccensis* | 2015 | Malaysia:Peninsula Malaysia:Johor | 51 | 50 | 0 | Oil palm plantation |
| 882899 | *Musa acuminata* subsp*. malaccensis* | 2015 | Malaysia:Peninsula Malaysia:Johor | 32 | 100 | 0 | Oil palm plantation |
| 928337 | *Musa acuminata* | 2016 | Malaysia:Peninsula Malaysia:Pahang | 36 | 40 | 0 | Jungle edge |
| 928360 | *Musa violascens* | 2016 | Malaysia:Peninsula Malaysia:Pahang | 13 | 0 | 0 | Roadside |
| 928429 | *Musa acuminata* | 2016 | Malaysia:Peninsula Malaysia:Johor | 18 | 40 | 0 | Oil palm plantation |
| 928500 | *Musa acuminata* subsp*. microcarpa* | 2016 | Malaysia: Peninsula Malyasia: Pahang | 50 | 0 | 0 | Roadside |
| 928717 | *Musa balbisiana* | 2015 | Vietnam:Lào Cai Province:Sa Pa | 40 | 20 | 0 | Jungle edge |
| 928728 | *Musa balbisiana* var*. balbisiana* | 2016 | Vietnam:Hà Giang Province | 58 | 0 | 0 | Jungle edge |
| 928739 | *Musa balbisiana* | 2016 | Vietnam:Hà Giang Province | 46 | 10 | 0 | Jungle edge |
| 928740 | *Musa balbisiana* var*. balbisiana* | 2016 | Vietnam:Hà Giang Province | 54 | 20 | 0 | Jungle edge |
| 944548 | *Musa velutina* | 2017 | Royal Botanic Gardens, Kew | NA | NA | NA | Botanical garden |
