# Supplementary material for: Seed Banks as Incidental Fungi Banks: Fungal Endophyte Diversity in Stored Seeds of Banana Wild Relatives
Source: Front Microbiol. 2021 Mar 22;12:643731. doi: 10.3389/fmicb.2021.643731 (PMC8024981; doi:10.3389/fmicb.2021.643731)
Supplement: Supplementary Table 2 — GenBank accession numbers for taxa used in the phylogenetic analysis. Accessions in bold were sequenced in this study. [file Table_2.docx]

**Supplementary Table 2.** GenBank accession numbers for taxa used in the phylogenetic analysis. Accessions in bold were sequenced in this study.

| Voucher | Species | EF1α | RPB1 | RPB2 |
| --- | --- | --- | --- | --- |
| NRRL 13412 | *Albonectria rigidiuscula (=Fusarium decemcellulare)* |  | JX171453 | JX171567 |
| NRRL 36160 | *Bisifusarium delphinoides (=Fusarium delphinoides)* | HM347134 | JX171535 | HM347219 |
| NRRL 20691 | *Bisifusarium dimerum (=Fusarium dimerum)* | EU926349 | JX171478 | JX171592 |
| NRRL 36168 | *Bisifusarium lunatum (=Fusarium lunatum)* | EU926291 | JX171536 | JX171648 |
| NRRL 20689 | *Bisifusarium nectrioides (=Fusarium nectrioides)* | EU926312 | JX171477 | JX171591 |
| NRRL 20711 | *Bisifusarium penzigii (=Fusarium penzigii)* | HM347132 | JX171482 | HM347217 |
| NRRL 36148 | *Cyanonectria buxi (=Fusarium buxicola)* |  | JX171534 | JX171647 |
| NRRL 13308 | *Fusarium acutatum* | AF160276 |  |  |
| NRRL 22152 | *Fusarium albidum* |  | JX171492 | JX171605 |
| NRRL 20459 | *Fusarium albosuccineum* |  | JX171471 | JX171585 |
| NRRL 25385 | *Fusarium anguioides* |  | JX171511 | JX171624 |
| NRRL 32997 | *Fusarium arcuatisporum* 'FIESC 7a' | GQ505624 |  | GQ505802 |
| NRRL 6227 | *Fusarium armeniacum* | HM744692 | JX171446 | JX171560 |
| NRRL 13818 | *Fusarium asiaticum* | AF212451 | JX171459 | JX171573 |
| NRRL 54939 | *Fusarium avenaceum* |  | JX171551 | JX171663 |
| NRRL 25410 | *Fusarium aywerte* |  | JX171513 | JX171626 |
| NRRL 25539 | *Fusarium babinda* |  | JX171519 | JX171632 |
| NRRL 25174 | *Fusarium beomiforme* |  | JX171506 | JX171619 |
| NRRL 31008 | *Fusarium brachygibbosum* |  | JX171529 | JX171642 |
| NRRL 43638 | *Fusarium brevicaudatum* 'FIESC 6a' | GQ505665 |  | GQ505843 |
| NRRL 13371 | *Fusarium buharicum* |  | JX171449 | JX171563 |
| NRRL 13829 | *Fusarium* cf. *compactum* |  | JX171460 | JX171574 |
| NRRL 25331 | *Fusarium circinatum* | KM231943 | JX171510 | JX171623 |
| NRRL 32871 | *Fusarium clavum* 'FIESC 5a' | GQ505619 |  | GQ505797 |
| NRRL 28577 | *Fusarium coffeatum* 'FIESC 28a' | GQ505603 |  | GQ505781 |
| NRRL 28387 | *Fusarium commune* |  | JX171525 | JX171638 |
| NRRL 36323 | *Fusarium compactum* 'FIESC 3a' | GQ505648 |  | GQ505826 |
| NRRL 13459 | *Fusarium concolor* | GQ505674 | JX171455 | JX171569 |
| NRRL 3020 | *Fusarium croceum* 'FIESC 10a' | GQ505586 |  | GQ505764 |
| InaCC F983 | *Fusarium cugenangense* | LS479756 | LS479559 | LS479307 |
| NRRL 25475 | *Fusarium culmorum* | AF212463 | JX171515 | JX171628 |
| NRRL 53998 | *Fusarium cyanostomum* | HM626647 | JX171546 | JX171658 |
| NRRL 29976 | *Fusarium domesticum* | EU926286 | JX171528 | JX171641 |
| NRRL 36401 | *Fusarium duofalcatisporum* 'FIESC 2a' | GQ505651 |  | GQ505829 |
| FocMal43 | *Fusarium duoseptatum* 'Race1' | LS479653 |  | LS479207 |
| NRRL 20697 | *Fusarium equiseti* | GQ505594 | JX171481 | JX171595 |
| NRRL 6548 | *Fusarium flagelliforme* 'FIESC 12a' | GQ505589 |  | GQ505767 |
| NRRL 25473 | *Fusarium flocciferum* |  | JX171514 | JX171627 |
| NRRL 28852 | *Fusarium fractiflexum* | AF160288 |  |  |
| NRRL 13566 | *Fusarium fujikuroi* | AF160279 | JX171456 | JX171570 |
| NRRL 45417 | *Fusarium gaditjirri* |  | JX171542 | JX171654 |
| NRRL 26131 | *Fusarium globosum* | AF160285 | KF466396 | KF466406 |
| NRRL 43635 | *Fusarium gracilipes* 'FIESC 13a' | GQ505662 |  | GQ505840 |
| NRRL 31084 | *Fusarium graminearum* | HM744693 | JX171531 | JX171644 |
| NRRL 20692 | *Fusarium graminum* |  | JX171479 | JX171593 |
| InaCC F820 | *Fusarium grosmichelii* 'Race1' | LS479810 |  | LS479364 |
| NRRL 32865 | *Fusarium guilinense* 'FIESC 21b' | GQ505614 |  | GQ505792 |
| NRRL 22945 | *Fusarium guttiforme* |  | JX171505 | JX171618 |
| NRRL 26417 | *Fusarium hainanense* 'FIESC 26a' | GQ505598 |  | GQ505776 |
| NRRL 20693 | *Fusarium heterosporum* |  | JX171480 | JX171594 |
| InaCC F866 | *Fusarium hexaseptatum* 'Race1' | LS479805 |  | LS479359 |
| NRRL 29889 | *Fusarium hostae* | AY329034 | JX171527 | JX171640 |
| NRRL 13379 | *Fusarium incarnatum* 'FIESC 23b' | GQ505591 |  | GQ505769 |
| NRRL 20433 | *Fusarium inflexum* | AF008479 | JX171469 | JX171583 |
| NRRL 43637 | *Fusarium ipomoeae* 'FIESC 1a' | GQ505664 |  | GQ505842 |
| NRRL 32175 | *Fusarium irregulare* 'FIESC 15a' | GQ505609 |  | GQ505787 |
| NRRL 20423 | *Fusarium lacertarum* | GQ505593 | JX171467 | JX171581 |
| NRRL 54940 | *Fusarium langsethiae* |  | JX171550 | JX171662 |
| NRRL 36372 | *Fusarium longifundum* 'FIESC 11a' | GQ505649 |  | GQ505827 |
| NRRL 13368 | *Fusarium longipes* |  | JX171448 | JX171562 |
| NRRL 31167 | *Fusarium luffae* 'FIESC 18a' | GQ505608 |  | GQ505786 |
| NRRL 54252 | *Fusarium lyarnte* |  | JX171549 | JX171661 |
| NRRL 25226 | *Fusarium mangiferae* | AF160281 | JX171509 | JX171622 |
| NRRL 26231 | *Fusarium miscanthi* |  | JX171521 | JX171634 |
| NRRL 43639 | *Fusarium multiceps* 'FIESC 19a' | GQ505666 |  | GQ505844 |
| MUCL 52574 | *Fusarium musae* | FN552086 |  |  |
| NRRL 22244 | *Fusarium nanum* 'FIESC 25a' | GQ505596 |  | GQ505774 |
| NRRL 54600 | *Fusarium nematophilum* |  | JX171552 | JX171664 |
| NRRL 25179 | *Fusarium nisikadoi* |  | JX171507 | JX171620 |
| NRRL 36452 | *Fusarium nurragi* |  | JX171538 | JX171650 |
| NRRL 54006 FocII5 | *Fusarium odoratissimum* 'TR4' | LS479644 | LS479459 | LS479198 |
| CAV300 | *Fusarium oxysporum* f*. cubense* 'TR4' | FJ664932 |  |  |
| NRRL 32864 | *Fusarium pernambucanum* 'FIESC 17a' | GQ505613 |  | GQ505791 |
| FocIndo25 | *Fusarium phialophorum* 'Race1' | LS479650 | LS479464 | LS479204 |
| NRRL 13714 | *Fusarium poae* |  | JX171458 | JX171572 |
| NRRL 22944 | *Fusarium proliferatum* |  | JX171504 | JX171617 |
| NRRL 28062 | *Fusarium pseudograminearum* | AF212468 | JX171524 | JX171637 |
| ATCC76244 | *Fusarium purpurascens* 'Race1' | LS479645 |  | LS479199 |
| NRRL 22901 | *Fusarium redolens* |  | JX171503 | JX171616 |
| NRRL 22134 | *Fusarium rusci* |  | JX171490 | JX171603 |
| NRRL 13999 | *Fusarium sacchari* |  | JX171466 | JX171580 |
| NRRL 22187 | *Fusarium sambucinum* |  | JX171493 | JX171606 |
| NRRL 20472 | *Fusarium sarcochroum* |  | JX171472 | JX171586 |
| NRRL 13402 | *Fusarium scirpi* |  | JX171452 | JX171566 |
| NRRL 36526 | *Fusarium setosum* |  | JX171539 | JX171651 |
| NRRL 26427 | *Fusarium* sp. | AF160286 |  |  |
| NRRL 25309 | *Fusarium* sp. | AF160284 |  |  |
| NRRL 25303 | *Fusarium* sp. | AF160283 |  |  |
| NRRL 34002 | *Fusarium* sp*.* 'FIESC 22a' | GQ505626 |  | GQ505804 |
| NRRL 20722 | *Fusarium* sp*.* 'FIESC 27a' | GQ505595 |  | GQ505773 |
| NRRL 5537 | *Fusarium* sp*.* 'FIESC 8a' | GQ505588 |  | GQ505766 |
| NRRL 29134 | *Fusarium* sp*.* 'FIESC 9a' | GQ505605 |  | GQ505783 |
| 836490-12 | *Fusarium* sp. OTU1 | **MW319605** |  |  |
| 880138-05 | *Fusarium* sp. OTU10 | **MW319601** |  |  |
| 836445-03 | *Fusarium* sp. OTU2 | **MW319595** |  |  |
| 880323-07 | *Fusarium* sp. OTU3 | **MW319620** |  |  |
| 880334-09 | *Fusarium* sp. OTU4 | **MW319629** |  |  |
| 880149-04 | *Fusarium* sp. OTU5 | **MW319604** |  |  |
| 836490-20 | *Fusarium* sp. OTU6 | **MW319587** |  |  |
| 836489-15 | *Fusarium* sp. OTU7 | **MW319636** |  |  |
| 836445-18 | *Fusarium* sp. OTU8 | **MW319589** |  |  |
| 880600-17 | *Fusarium* sp. OTU9 | **MW319588** |  |  |
| NRRL 3229 | *Fusarium sporotrichioides* | HM744665 | JX171444 | JX171558 |
| NRRL 20429 | *Fusarium stilboides* |  | JX171468 | JX171582 |
| NRRL 22016 | *Fusarium subglutinans* | HM057336 | JX171486 | JX171599 |
| NRRL 13384 | *Fusarium sublunatum* |  | JX171451 | JX171565 |
| NRRL 34004 | *Fusarium sulawesiense* 'FIESC 16a' | GQ505628 |  | GQ505806 |
| NRRL 34005 | *Fusarium tanahbumbuense* 'FIESC 24a' | GQ505629 |  | GQ505807 |
| InaCC F956 | *Fusarium tardichlamydosporum* 'Race1' | LS479727 | LS479532 | LS479278 |
| NRRL 22045 | *Fusarium thapsinum* |  | JX171487 | JX171600 |
| NRRL 54149 | *Fusarium torreyae* |  | JX171548 | JX171660 |
| NRRL 22748 | *Fusarium torulosum* |  | JX171502 | JX171615 |
| NRRL 25481 | *Fusarium tricinctum* |  | JX171516 | JX171629 |
| NRRL 22196 | *Fusarium venenatum* |  | JX171494 | JX171607 |
| NRRL 22566 | *Fusarium verrucosum* |  | JX171500 | JX171613 |
| NRRL 20956 | *Fusarium verticillioides* |  | JX171485 | JX171598 |
| NRRL 25486 | *Fusarium xylarioides* |  | JX171517 | JX171630 |
| NRRL 22316 | *Geejayessia atrofusca (=Fusarium staphyleae)* |  | JX171496 | JX171609 |
| NRRL 22465 | *Geejayessia zealandica (=Fusarium zealandicum)* |  | JX171498 | JX171611 |
| NRRL 20438 | *Neocosmospora ambrosia (=Fusarium ambrosium)* | AF178332 | JX171470 | JX171584 |
| NRRL 43529 | *Neocosmospora falciformis (=Fusarium falciforme)* | EF452965 | JX171541 | JX171653 |
| NRRL 22090 | *Neocosmospora illudens (=Fusarium illudens)* | AF178326 | JX171488 | JX171601 |
| NRRL 45880 | *Neocosmospora pisi (=Fusarium solani* f. sp. *pisi)* |  | JX171543 | JX171655 |
| NRRL 22632 | *Neocosmospora plagianthi (=Fusarium plagianthi)* | AF178354 | JX171501 | JX171614 |
| NRRL 22436 | *Neocosmospora vasinfecta (=Fusarium neocosmosporiellum)* | AF178348 | JX171497 | JX171610 |
| NRRL 22276 | *Neocosmospora phaseoli (=Fusarium phaseoli)* | EF408415 | JX171495 | JX171608 |
| NRRL 31041 | *Neocosmospora phaseoli (=Fusarium virguliforme)* |  | JX171530 | JX171643 |
| NRRL 20485 | *Neonectria coccinea* |  | JX171474 | JX171588 |
| NRRL 22505 | *Neonectria neomacrospora (=Cylindrocarpon cylindroides)* |  | JX171499 | JX171612 |
| NRRL 20846 | *Rectifusarium ventricosum (=Fusarium ventricosum)* |  | JX171484 | JX171597 |
