# Supplementary material for: Seed Banks as Incidental Fungi Banks: Fungal Endophyte Diversity in Stored Seeds of Banana Wild Relatives
Source: Front Microbiol. 2021 Mar 22;12:643731. doi: 10.3389/fmicb.2021.643731 (PMC8024981; doi:10.3389/fmicb.2021.643731)
Supplement: Supplementary Table 3 — Summary of OTUs which were also found on the unsterilised seed surface. [file Table_3.docx]

**Supplementary Table 3**. Summary of OTUs which were also found on the unsterilised seed surface.

| Species | OTU | Count |
| --- | --- | --- |
| Otu22 | *Fusarium concentricum* | 5 |
| Otu52 | Capnodiales sp. | 1 |
| Otu6 | *Lasiodiplodia citricola* | 2 |
| Otu8 | Nectriaceae sp. | 1 |
| Otu176 | Ascomycota sp. | 1 |
| Otu44 | *Neofusicoccum parvum* | 1 |
| Otu99 | Xylariales sp. | 1 |
| Otu13 | *Aspergillus flavus* | 1 |
| Otu27 | *Aspergillus niger* | 7 |
| Otu196 | *Penicillium meleagrinum* var. *viridiflavum* | 1 |
| Otu26 | Capnodiales sp. | 2 |
| Otu62 | *Talaromyces ricevillensis* | 1 |
| Otu166 | Hypocreales sp. | 1 |
| Otu96 | Ascomycota sp. | 1 |
| Otu36 | *Penicillium solitum* | 2 |
